# Estimating Driving Fatigue at a Plateau Area with Frequent and Rapid Altitude Change

**DOI:** 10.3390/s19224982

**Published:** 2019-11-15

**Authors:** Fan Wang, Hong Chen, Cai-hua Zhu, Si-rui Nan, Yan Li

**Affiliations:** 1 School of Highway, Chang’an University, Xi’an 710064, China; wfssjj@chd.edu.cn (F.W.); chh@gl.chd.edu.cn (H.C.); zhucaihua@chd.edu.cn (C.-h.Z.); 2 State Key Laboratory of Road Engineering Safety and Health in Cold and High-altitude Regions, China Communications Construction Company First Highway Consultants Co., LTD, Xi’an 710075, China; 3 School of Transportation, Southeast University, Nanjing 211189, China; 230198689@seu.edu.cn

**Keywords:** driving fatigue, rapid altitude change, correction factor, heart rate variability, blinking frequency, Qinghai-Tibet Plateau

## Abstract

Due to the influence of altitude change on a driver’s heart rate, it is difficult to estimate driving fatigue using heart rate variability (HRV) at a road segment with frequent and rapid altitude change. Accordingly, a novel method of driving fatigue estimation for driving at plateau area with frequent altitude changes is proposed to provide active safety monitoring in real time. A naturalistic driving experiment at Qinghai-Tibet highway was conducted to collect drivers’ electrocardiogram data and eye movement data. The results of the eye movement-based method were selected to enhance the HRV-based driving fatigue degree estimation method. A correction factor was proposed to correct the HRV-based method at the plateau area so that the estimation can be made via common portable devices. The correction factors for both upslope and downslope segments were estimated using the field experiment data. The results on the estimation of revised driving fatigue degree can describe the driver’s fatigue status accurately for all the road segments at the plateau area with altitudes from 3540 to 4767 m. The results can provide theoretical references for the design of the devices of active safety prevention.

## 1. Introduction

### 1.1. Background

One of the greatest risks of driving at high altitude area is altitude sickness [1], which is caused by rapid exposure to low amounts of oxygen at high elevation [2]. Drivers with altitude sickness may experience a longer reaction time, misjudgments, and even faulty operation [3]. Thus, altitude sickness must be detected as soon as possible to prevent more serious consequences once the driver has related symptoms. Early symptoms of altitude sickness may include headaches, fatigue, and dizziness [4]. Among them, driving fatigue degree (DFD) is considered to be the most direct indicator of altitude sickness [5] and can be utilized as a reliable source in the field of active safety prevention [6,7]. As symptoms may develop much more rapidly than usual when a road segment has a rapid and constant change in elevation [8], these road segments become very dangerous for drivers, especially for non-local drivers that are new to plateau areas. The safety concerns on these road segments have been improved recently with demands for the construction of high-level highways in these areas.

There are two kinds of commonly used methods to estimate DFD, namely eye movement-based [9] and heart rate-based [10] methods. The data used to estimate DFD needs to be precise, easily accessed, and have little influence on drivers [11]. Therefore, the usage of sensors, like an eye tracker, is not suitable for practical applications. A driver’s real-time heart rate can be obtained from a heart rate belt or wristband, which has little influence on drivers. However, heart rate is significantly affected by the change of elevation [12], i.e., this method will be invalid for road segments with rapid and frequent altitude changes. Therefore, a novel DFD estimation method for road segments with rapid altitude change is urgently needed for possible application of active safety prevention in these areas.

### 1.2. Literature Review

The harsh external driving environment at high altitude areas, such as low oxygen content and temperature, makes it the most critical risk factor for driving safety [13]. Drivers are prone to fatigue in such an environment. The results of field experiments at the Qinghai-Tibet plateau indicated that drivers at a higher altitude are more prone to fatigue, especially for non-local drivers [14]. Thus, the threshold for fatigue in high altitude areas is much lower than that in low altitude areas. According to the severity of fatigue, DFD can be divided into mild, moderate, or severe fatigue [15]. Drivers with a severe fatigue status may have symptoms of decreased awareness, rapid heartbeat, and double vision, which may lead to traffic collisions [16]. As DFD is significantly affected by the change in altitude, DFD at road segments with rapid altitude will be influenced by both altitude and driving duration. If inputs for the DFD estimation method include variables that are affected by altitude, this method cannot be utilized at these road segment directly.

The estimation of DFD can be either subjective or objective. The subjective estimation of DFD is based on post-evaluations of drivers [17]. The drivers are asked to fill a questionnaire form, which may include certain indicators of subjective perception on driving fatigue [18]. The DFD can then be estimated based on the researcher’s experience. This method is simple to operate and easy to implement, but has low reliability. The objective estimations of DFD can use various data collected from drivers. These inputs include, but are not limited to, physiological indicators, naturalistic driving behaviors, and vehicle behaviors. Physiological indicators are considered to be the best measurements for driving fatigue and include electroencephalogram (EEG) [19], electrocardiogram (ECG) [20], electromyography (EMG) [21], heart rate [22], eye gaze distribution [9], saccade range, and blink frequency [23]. As drivers typically do not wear sensors, driving behaviors, such as eye closure duration [24] and yawning frequency [25], can only be extracted from videos captured by monitoring cameras. Researchers must estimate driving fatigue status based on these driving behaviors. The vehicle behaviors measured can be hand pressure on the wheel [26], steering angle [27], deviation from the lane [28], etc. The driver’s physiological indicators collected in the field driving environment have a better representation on real driving fatigue state, but a safer choice may be a driving simulator [29]. When data needs to be processed in real-time, sensors must be powerful enough to process data within a limited time and be portable enough to minimize impact on participants [30]. The DFD can be either obtained based on the statistics of serval indicators [16,31] or from data processing algorithms. The data processing algorithms that have been used in related researches include artificial neural network [32], Bayesian network [33], spectrum analysis [34], wavelet transforms [35], Gaussian process mixture models [36], accident tree method [37], multivariate Tobit model [38], and support vector machine [39].

Little research has focused on the estimation of DFD at a road segment with constant and rapid altitude change for the purpose of active safety prevention. Three gaps make DFD obtained from existing research invalid when applied to the real driving process. Firstly, the DFD estimated by a driver’s heart rate and blood oxygen content is person specific, which can hardly be utilized as a universal standard. Secondly, regular drivers have little chance to wear a portable eye tracker to estimate DFD. Instead of estimation in real-time, DFD estimated in this way is usually processed after the experiment. Thirdly, altitude changes have significant influences on a driver’s heart rate and heart rate variability (HRV) characteristics, which make them difficult to be used in the estimation of DFD in these areas.

### 1.3. Objective and Contributions

The objective of this study was to propose a method to estimate DFD at a plateau area with constant and rapid altitude change through a naturalistic driving experiment along the Qinghai-Tibet Highway. The major contributions of this study are two-fold: (1) describing driving fatigue characteristics at a plateau area with constant and rapid altitude change using naturalistic driving data and (2) enhancing the HRV-based DFD estimation method using naturalistic eye movement data. The revised model can be utilized to establish the DFD at a plateau area with constant and rapid altitude change with wearable sensors.

The remainder of this paper is organized as follows: Section 2 introduces the experimental design and the data collected. Section 3 proposes a revised DFD estimation method for a plateau area with rapid altitude changes. The driving fatigue characteristics and related DFD estimation models at a plateau area are verified using a driver’s naturalistic driving physiological data in Section 4. Section 5 discusses the reliability of the proposed model and Section 6 concludes the paper.

## 2. Experimental Design

### 2.1. Experimental Environment

**Figure 1:**
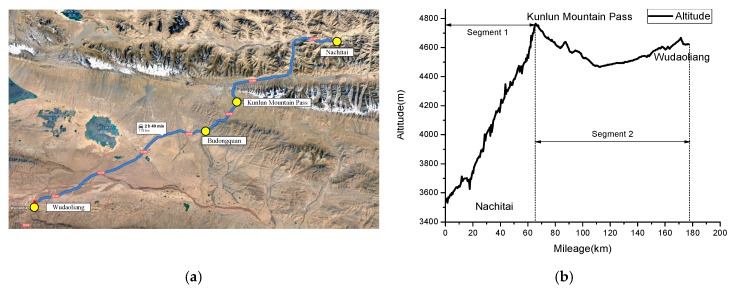
(**a**) Layout of the experimental route; and (**b**) altitudes along the experimental route.

The road segment between Nachitai (Distance: K2828, Altitude: 3540 m) and Wudaoliang (Distance: K3006, Altitude: 4663 m) of the Qinghai-Tibet Highway, belonging to China National Highways 109 (G109), was selected to collect field drivers’ physiological data (see Figure 1a). The Qinghai-Tibet Highway is a two-lane highway with a speed limit of 60 km/h. The altitude between Nachitai and the Kunlun Mountain Pass (Distance: K2898, the highest altitude) increases continually from 3540 m to 4768 m. The altitude after the Kunlun Mountain Pass first decreases to 4476 m at Budongquan and then increases to 4663 m at Wudaoliang. The altitudes of the tested route are shown in Figure 1b. The surface of the selected road segment was smooth and road markings were clear. The driving duration of a one-way trip was approximately 60 min from Nachitai to the Kunlun Mountain Pass and 190 min from Nachitai to Wudaoliang.

### 2.2. Sample Population

Because of the extreme road environmental conditions at the plateau area, only 13 drivers with driving experience of no less than 6 years were selected for field experiments. Ten drivers were local male drivers. The other three drivers (two male and one female) were new to the Qinghai-Tibet plateau. All participants were between 36 and 51 years of age, free from any medication, serious head injuries, or hearing impairments, and had normal or corrected-to-normal vision. Only one local driver was older than 50 (age 51) at the time of experiment. The *t*-test for these samples showed that there was no significant difference between the physiological indicators collected from non-local drivers and local drivers. Although the heart rate indicators of non-local drivers were significantly higher than those of local drivers, they represented a similar trend in describing driving fatigue. In this way, all available samples from these 13 drivers were classified into the same data set.

Each participant gave written consent before the experiment began and participants were free to leave the experiment in any stage if they felt uncomfortable. This study received ethics approval from the State Key Laboratory of Road Engineering Safety and Health in Cold and High-Altitude Regions of CCCC First Highway Consultants Co., Ltd. and the Ethics Committee of Chang’an University.

### 2.3. Equipment Selection and Installation

**Figure 2:**
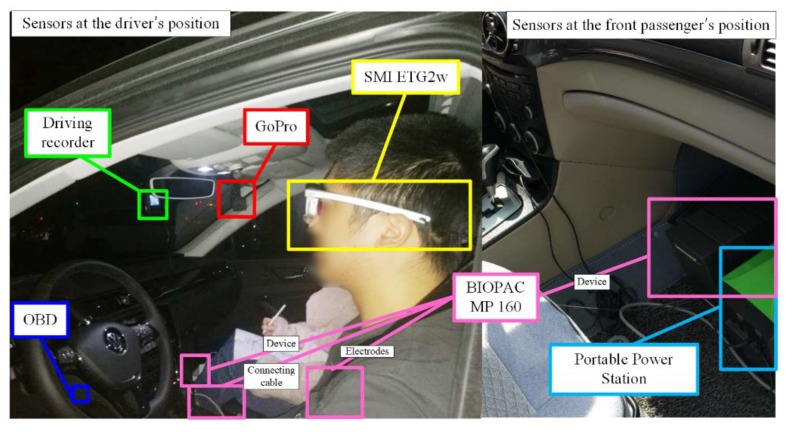
Equipment installation in the experiment.

The commonly used DFD estimation methods need heart rate/electrocardiogram or blinking frequency data as inputs [9,10]. The wearable sensors were selected to obtain these physiological data with minimal influence on participants. The selected sensors were the BIOPAC MP 160 physiological data acquisition system and SMI ETG2w eye-tracking glasses. Meanwhile, other sensors, such as OBDs (on-board diagnostics), driving recorder, and GoPro video cameras, were also installed on the test vehicle to collect dynamic vehicle information and other naturalistic driving information. All the sensors were strictly tested to ensure that there was no significant influence on driving actions. The equipment installations on the test vehicle is illustrated in Figure 2.

**Figure 3:**
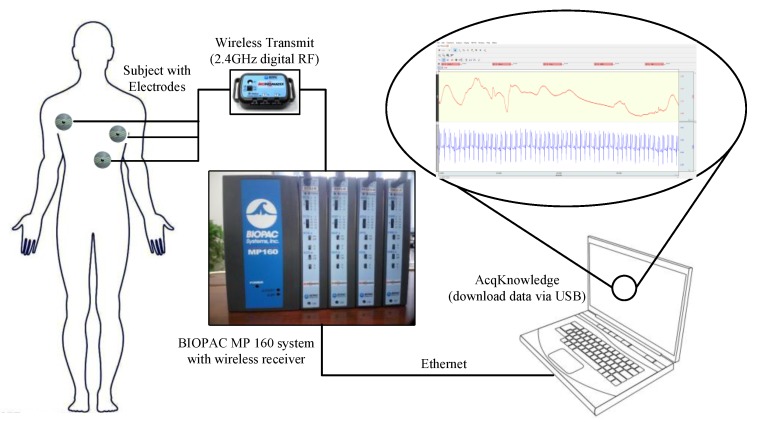
The installation and data processing of the BIOPAC MP 160 data acquisition system.

The BIOPAC MP 160 physiological data acquisition system has 16 channels with the capability to record reproducible ECG, EEG, EMG, blood pressure, blood oxygen content, and more data. The collected data can be measured using related detachable modules on the MP160 system. The micro-electrodes were attached to the modules of MP 160 to record ECG data with a sampling rate of 400 kHz (aggregated). The placement of the three electrodes needed by the MP 160 system is shown in Figure 3. All the recorded signals from the sensors were sent to a laptop using Ethernet and were processed with AcqKnowledge software. The installation of the MP 160 system and user interface of AcqKnowledge software is illustrated in Figure 3.

**Figure 4:**
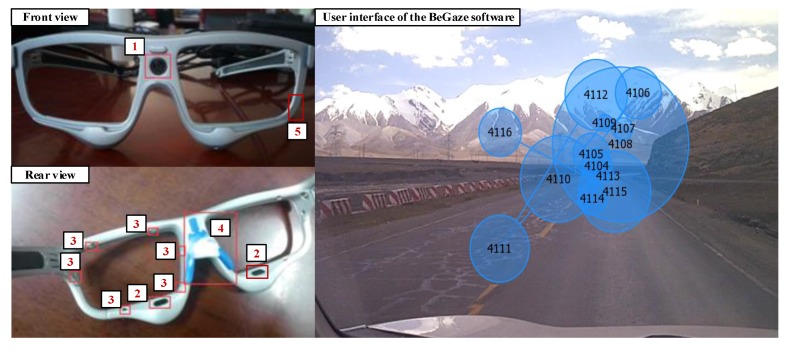
Sensors on the SMI ETG2w system and data processing interface.

The SMI ETG 2w system can record a driver’s natural gaze behavior in real time with adequate robustness, mobility, and ease of use. As shown in Figure 4, the SMI ETG 2w uses two high-speed eye cameras with of infrared detection technology (number 2 in Figure 4) as a critical sensor for blinking frequency detection. Meanwhile, a high-resolution scene camera (number 1 in Figure 4) and one microphone (number 5 in Figure 4) were installed to collect field information during the experiment. Twelve fill-in light-emitting diode (LED) lights (number 3 in Figure 4) constantly provide enough light during the sensing process and the nose rest (number 4 in Figure 4) can provide a comfortable wear experience. Non-shaded lenses or assorted myopia-correcting lenses could be selected to fit all participants. The SMI ETG 2w system works with an Android smartphone during the recording process. The data can be further processed using SMI BeGaze software.

### 2.4. Experimental Process

The experiments were carried out from 16 to 20 June 2015 and 23 to 25 June 2015. During the experiments, the weather was good and traffic volume was relatively low. All participants were asked to rest well the previous night. Tobacco, wine, coffee, and drug consumption was forbidden on the previous day. Other participants onboard were asked to keep silent during the driving process to avoid distractions. Other measures to improve accuracy, such as wearing loose clothes, were also considered in the experiments.

All drivers started the experiment from the experimental base at Nachitai. The terminal points were decided based on drivers’ physical conditions. Before the experiment began, a training section was carried out to reduce the volatility of indicators in the first 10 min. If the driver was in a poor health condition, he/she might return from the Kunlun Mountain Pass or abandon the experiment. Otherwise, the drivers were to drive to Wudaoliang and return to the experimental base. When the participants arrived at the terminal point, they had a rest for half an hour and then continued the experiment. All local drivers finished the whole experiment route. Two non-local drivers stopped the experiment at Kunlun Mountain Pass and drove back. One male driver abandoned the experiment because of his health considerations.

### 2.5. Measures of Data Reliability and Driving Safety

The naturalistic driving video and the driver’s post-test evaluation were used to determine their driving fatigue status. All drivers needed to fill out a questionnaire about their driving fatigue status during the experimental process. Driving fatigue was divided into four levels, which were no fatigue, mild fatigue, moderate fatigue, and severe fatigue. When there was no fatigue, the driver feels awake, alert, and attentive. The driver will feel like they are not in the best state but can keep focused when in a mild fatigue state. A driver in a moderate fatigue state may have delayed reactions and have difficulty keeping alert. When in a severe fatigue state, the driver will feel very tired and react slowly to traffic events [40]. All drivers’ driving fatigue states were estimated using these evaluation criteria and linked to heart rate and altitude.

Safety was always the first concern in our experiment. Realizing driving in a severe fatigue state will increase driving risk significantly, we asked participants in the front passenger position to pay attention to the driver’s fatigue state and surrounding traffic state. When the driver was in a severe fatigue state with bad traffic conditions, such as a high traffic volume or extreme road incline, they could halt the experiment until it was safe to proceed.

## 3. Revised DFD Estimation Method

### 3.1. Process for Revision of DFD Estimation

Both HRV data and eye blinking frequency can be utilized to estimate driving fatigue at road segments with low altitude or without frequent altitude change. However, only the estimation using blinking frequency can obtain a reasonable estimation of driving fatigue status for road segments with frequent altitude change. Considering only heart rate data is available for real-time driving fatigue estimation, the HRV-based DFD estimation model needs to be revised. In this way, revision of HRV-based DFD estimation can choose the results of the blinking frequency-based model as a reference. This revision will first scale both data into same range and then use an altitude-based correction factor to revise the HRV-based estimation according to the blinking frequency-based estimation.

### 3.2. Comparison of DFD Estimation Methods

The DFD can be calculated using either HRV or eye blinking data. When a driver goes into the fatigue state, the eye will blink spontaneously to keep the cornea moist. Thus, increased spontaneous blink frequency and decreased heart rate are characterized as drowsiness [5]. The HRV-based DFD estimation methods uses the heartbeat intervals (also known as the R-R intervals) as the input. When an electrocardiogram (ECG) is available, the R-R intervals are measured as the time difference between adjacent wave peaks. The variation coefficient of the R-R interval (RRVC) can be obtained from Equation (1). As current driving fatigue is the combination of driving fatigue and original fatigue, the differences in the RRVC between various states can be utilized to measure driving fatigues in various states, which are given by Equations (2)–(4). Then, the DFD can be calculated by Equation (5). Although the heart rate of each driver varies, the HRV parameters has a similar pattern [32]. Thus, the average value of the HRV parameters from all the participants was used to calculate the HRV-based DFD.

(1) $$RRVC=\frac{SDNN}{M},$$

where, SDNN is the standard deviation of the driver’s R-R interval series, and M is the average R-R interval.

(2) $$BDF=RRVC_{initial}-RRVC_{static}=\frac{SDNN_{initial}}{M_{initial}}-\frac{SDNN_{static}}{M_{static}},$$

(3) $$FC_{i}=RRVC_{i}-RRVC_{static}=\frac{SDNN_{i}}{M_{i}}-\frac{SDNN_{static}}{M_{static}},$$

(4) $$DFC_{i}=RRVC_{i}-RRVC_{initial}=\frac{SDNN_{i}}{M_{i}}-\frac{SDNN_{initial}}{M_{initial}},$$

(5) $$DFD_{i}=\frac{RRVC_{i}-RRVC_{initial}}{RRVC_{initial}-RRVC_{static}}=\frac{\frac{SDNN_{i}}{M_{i}}-\frac{SDNN_{initial}}{M_{initial}}}{\frac{SDNN_{initial}}{M_{initial}}-\frac{SDNN_{static}}{M_{static}}},$$

where, *BDF* is the driving fatigue before the experiment, *FC_i_* is the cumulative fatigue at the ith time interval, *i* ∈ [1, *n*], *DFC_i_* is the cumulative driving fatigue at the ith time interval, *DFD_i_* is the driving fatigue degree at the ith time interval, *RRVC_i_* is the variation coefficient of the R-R intervals at the ith time interval, $RRVC_{initial}$ is the initial variation coefficient of the R-R intervals, and $RRVC_{static}$ is the variation coefficient of the R-R intervals when the driver is in a peaceful state (such as when sitting still).

### 3.3. Revised DFD Estimation for a Segment with Constant Altitude Change

The blinking-based DFD needs to be scaled to have the same measurement as HRV-based DFD in the DFD revised algorithm. If the initial values of these DFDs are equal, a correction factor *δ* could be introduced to modify the HRV-based DFD to fit the actual pattern using Equation (6). Because altitude has the opposite influence on upslope and downslope segments, the correction factor *δ* should be calibrated separately:

(6) $$rDFD_{i}=\delta (h)\cdot DFD_{i},$$

where, *rDFD_i_* is the revised DFD at the ith time interval, *h* is the altitude, and *δ*(*h*) is the altitude-related correction factor, which is a dimensionless variable. The correction factor for upslope road segment and downslope road segment should be calibrated separately.

## 4. Results

### 4.1. Driving Fatigue Characteristics on the Qinghai-Tibet Highway

**Figure 5:**
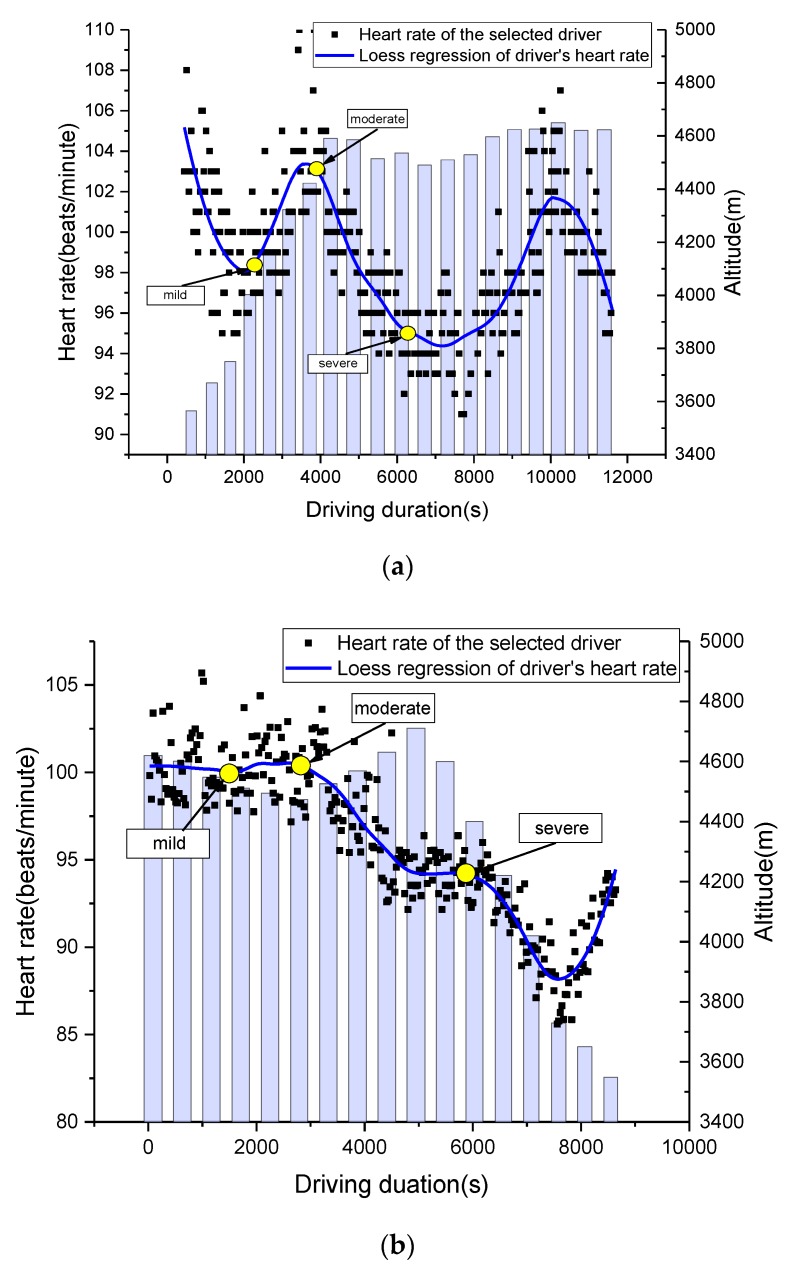
Variations in heart rate with altitude and driving duration. (**a**) Upslope trip and (**b**) downslope trip.

As heart rate data varies from person to person, the heart rate data of one local driver that had a similar pattern with the others was selected for further analysis. As shown in Figure 5, altitude has a more significant influence on heart rate when the altitude is above 4500 m. The approximate start time of each driving fatigue status, based on the driver’s evaluation, is also marked in Figure 5. Although the variations in heart rate can represent driving fatigue status at a road segment with slight altitude change [10], it is difficult to locate the change in driving fatigue status when the altitude changes constantly and rapidly. Besides the beginning and ending periods when heart rate has significant variations, the heart rate is basically represented by an “S shape”.

### 4.2. Verifications of DFD Estimation Methods

**Figure 6:**
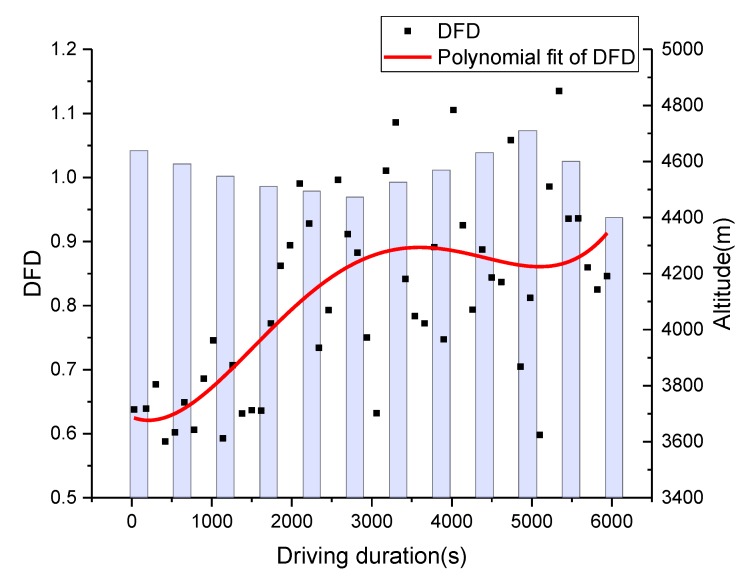
Variations of DFD with altitude and driving duration of segment 2.

**Figure 7:**
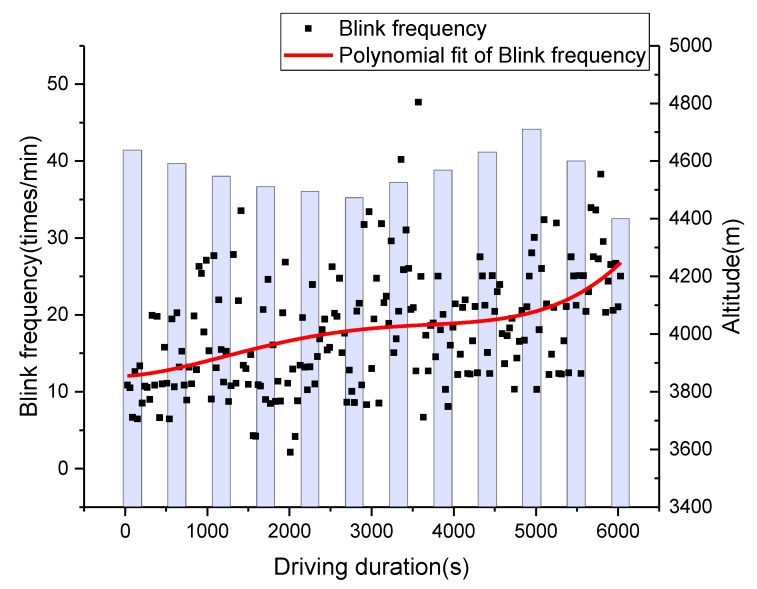
Variations in blinking frequency with altitude and driving duration of segment 2.

As shown in Figure 1b, the experiment route can be divided into two road segments: Nachitai-Kunlun Mountain Pass (segment 1), with constant and rapid altitude change, and Kunlun Mountain Pass-Wudaoliang (segment 2), with little altitude change. The DFD estimation methods were tested using filed driving data from the road segment from Wudaoliang to Kunlun Mountain Pass, which is the first part of the return trip. The average value from all drivers was utilized to calculate the DFD. The comparisons of the DFD estimations using HRV and blinking frequency are shown in Figure 6 and Figure 7, respectively. To minimize stochastic deviations of raw data in the estimation process, heart rate data was aggregated by 2-min time intervals. The unit of blinking frequency is times/min. The polynomial fittings (cubic fitting) to the original data series were performed to reveal their trends.

The results of both methods indicated that the drivers’ DFD represents an S-shape curve at the road segment with slight altitude change. Based on the drivers’ post-evaluation on severity of DFD, the times of mild, moderate, and severe driving fatigue were around 1500, 3000, and 6000 s, respectively. When driving fatigue state changed, an inflection point appeared on DFD curves in results from both methods.

According to the relationship between driving fatigue and driving duration, as shown in Figure 6 and Figure 7, driving fatigue rose very fast at the beginning of the return trip because the driver’s physical function had to adjust quickly to the level required to perform driving tasks in the plateau’s environment. With a massive consumption of energy, the driving fatigue increase was rapid. The increase in DFD was slower after reaching the moderate fatigue point when the driver’s physical function reached a balanced point. With the continuous increase in driving fatigue, the balance point of a driver’s physical function would be broken at the severe fatigue point, when DFD would increase quickly. Therefore, the inflection points of the DFD curves can be defined as the breakpoints of driving fatigue states.

**Figure 8:**
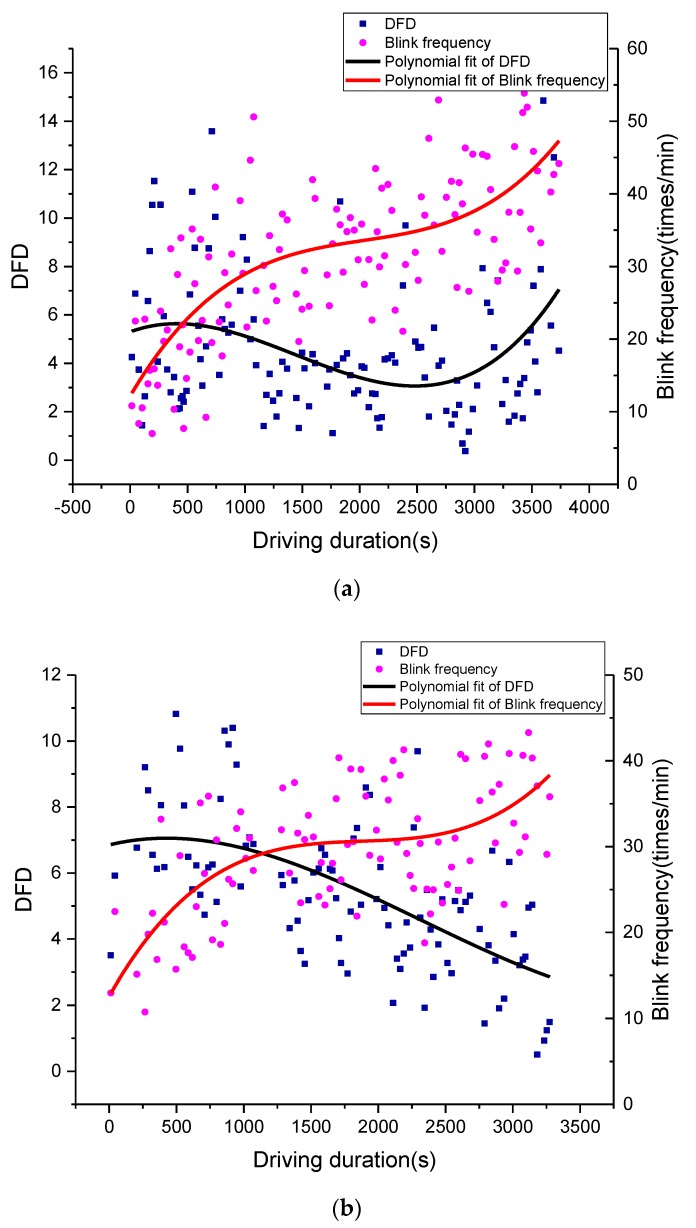
Variations in DFDs with altitude and driving duration of segment 1: (**a**) upslope road segment and (**b**) downslope road segment.

As heart rate is significantly affected by altitude change, the HRV-based DFD had significant differences compared to the DFD estimated by blinking frequency at the road segment with constant altitude change (see Figure 8). It is worth noting that the HRV-based DFD had a downward trend during the time interval from 1500 to 3000 s, which didn’t match the actual situation. However, blinking frequency-based DFD had a similar pattern with the actual situation at segment 2. In this way, the blinking frequency-based DFD data was selected as the base curve to modify the HRV-based DFD algorithm.

### 4.3. Revised DFD Estimation for a Road Segment with Constant Altitude Change

**Figure 9:**
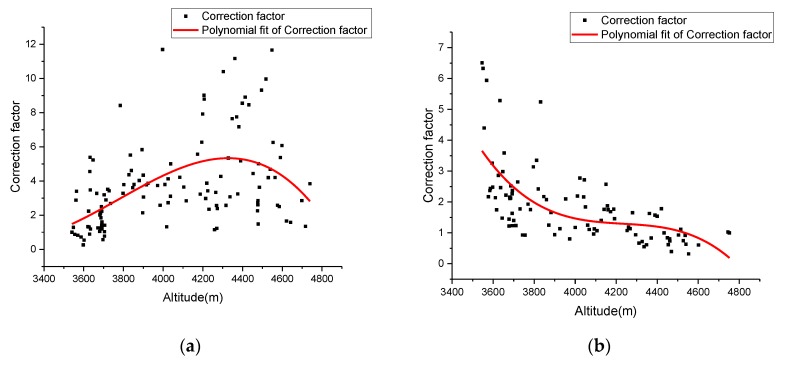
Variations in correction factor *δ* for segment 1: (**a**) upslope road segment and (**b**) downslope road segment.

**Figure 10:**
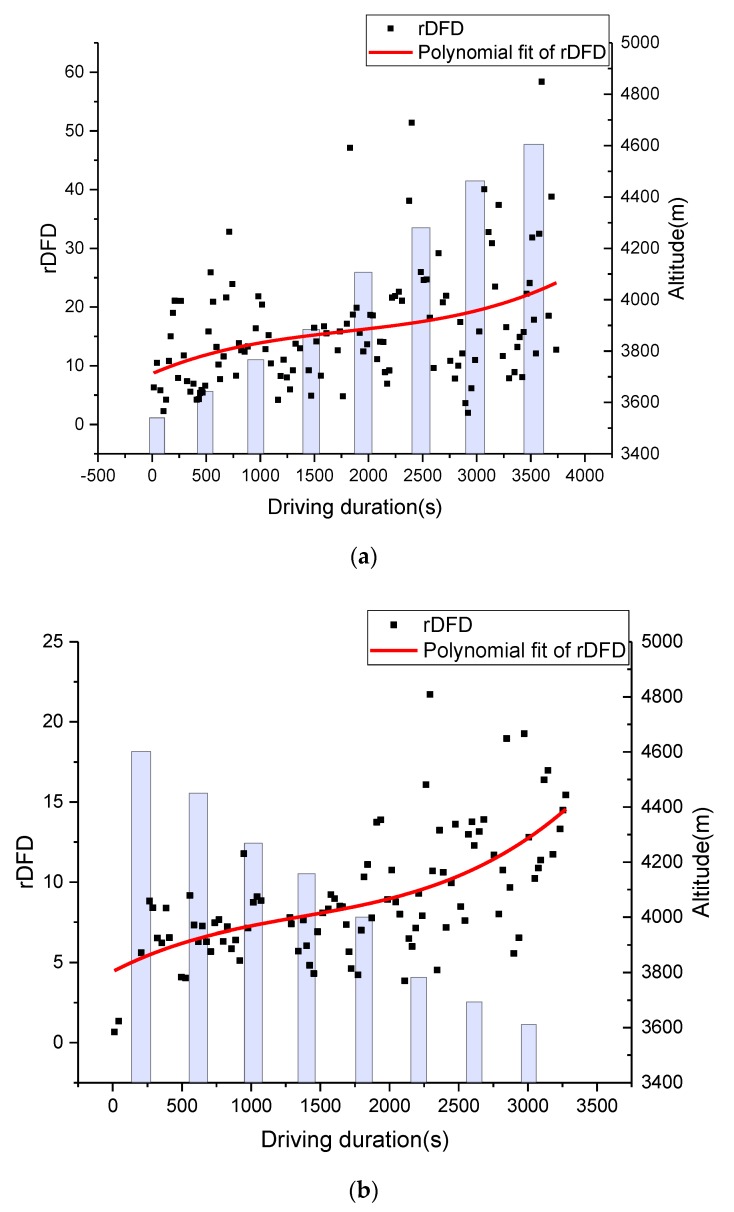
Variations in rDFDs with altitude and driving duration of segment 1: (**a**) upslope road segment and (**b**) downslope road segment.

Because altitude has an opposite influence on upslope and downslope segments, the correction factor *δ* of different segment types was calibrated separately. As shown in Figure 9, results of the calibrations on correction factor functions for both upslope and downslope segments were determined by Equations (7) and (8). Then, we can obtain revised DFD (rDFD) using Equations (9) and (10). The variations in rDFD with altitude and driving duration of segment 1 are illustrated in Figure 10. The rDFD can be utilized to estimate driving fatigue at a road segment with constant altitude change.

(7) $$\delta_{u}=366.711-0.302h+8.169\times 10^{-5}h^{2}-7.209\times 10^{-9}h^{3}.$$

(8) $$\delta_{d}=493.703-0.348h+8.219\times 10^{-5}h^{2}-6.473\times 10^{-9}h^{3}.$$

(9) $$rDFD_{u}=8.658+0.008t-3.173\times 10^{-6}t^{2}+5.850\times 10^{-10}t^{3}.$$

(10) $$rDFD_{d}=4.436+0.004t-2.173\times 10^{-6}t^{2}+5.348\times 10^{-10}t^{3}.$$

## 5. Discussion

Although a training section was conducted before the experiments, nearly all the drivers still had an “excitement period” at both the beginning and ending of the experiments. As shown in Figure 5, the drivers’ heart rates during these periods was unreasonably high. Considering this, the data for first and last 1000 s was removed to have better descriptions of driving fatigue. Then, the estimated DFD represented similar patterns to the low altitude areas.

The heart rate data was aggregated to acquire statistical indicators, such as SDNN. The length of time intervals also impacted the estimation of DFD. If the time interval is too short, SDNN may have greater randomness, which makes the change in DFD not significant. Meanwhile, a longer time interval will lose characteristics of heart rate variability, which may decrease the accuracy of the estimation. As shown in Table 1, the significance F (sig.) and F value of the estimated DFD were used to find the appropriate time interval. In statistics, the significance F is the probability that the null hypothesis in the proposed model cannot be rejected. And the F value is a ratio computed by dividing the mean regression sum of squares by the mean error sum of squares. The smaller the significance F (should be less than 0.05) and F value are, the better performance will be. With the considerations of both measures, the time interval was chosen as 2 min.

As shown in Figure 10, rDFD matched the patterns of DFD estimated by blinking frequency. Taking the downslope segment as an example, a driver’s heart rate will gradually decrease to a normal level as they can get more oxygen. The variations in heart rate will cause a decrease in driving fatigue when it is estimated using the traditional HRV-based method. As driving fatigue is a continuous accumulation process, driving fatigue will not represent a decreasing trend. As shown in Figure 10, rDFD can represent the continuous accumulation of driving fatigue with a lower growth rate, which will be a better estimation for driving fatigue at these road segments. As shown in Table 2, the sum of squares of difference between the compared curves, significance F (should be less than 0.05, the null hypothesis was there is no significant relationship between these two curves), and coefficient of determination (R^2^) were selected to describe the relationship between the compared algorithms. The results of the F-test indicated that the proposed algorithm was more related to the blinking frequency-based algorithm, i.e., has a better description of driving fatigue for road segments with constant and rapid altitude changes.

Compared with DFD in low altitude areas, the drivers tended to get fatigued more quickly. The higher the altitude was, the faster that driver became fatigued. As the results indicated in Figure 10, a driver will become fatigued faster at an upslope segment, and vice versa. The influence of altitude change seems to be the critical influencing factor on driving fatigue, i.e., drivers took less time to become fatigued at the upslope segment than they were at segment 2.

**Figure 11:**
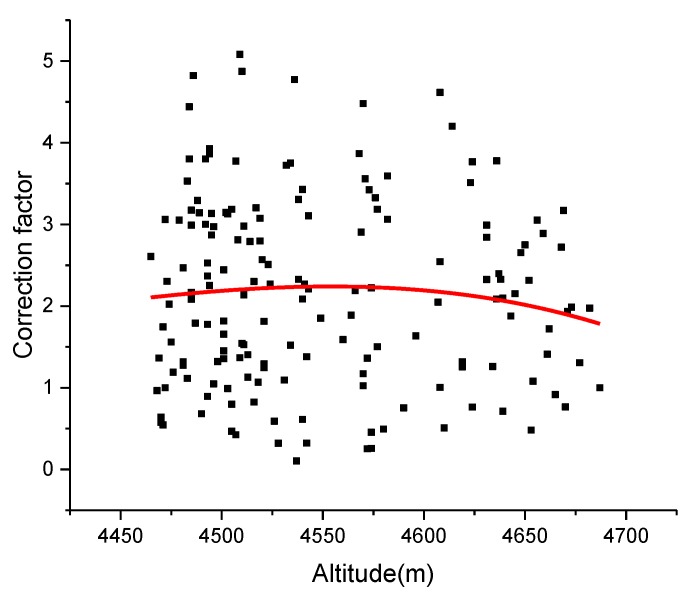
Variations in correction factor δ with altitude of segment 2.

As shown in Figure 11, the rDFD estimation method was also tested at road segment 2, where there is little altitude change. The correction factors at road segment 2 were also estimated using the proposed method. The results indicated that the change in altitude has very little influence on the correction factor when altitude is lower than 4550 m. The correction factor has a slight decline with the increase in altitude when the altitude is higher than 4550 m. The F-test between correction factor and altitude obtained a significance F of 0.78483 and an F value of 0.35604, which means that the null hypothesis of no relationship between test sets should be accepted. In this way, the overall oscillation of scale factor is relatively small, which verifies the validity of the proposed model.

## 6. Conclusions

The driving fatigue in the plateau area with rapid altitude change was estimated using drivers’ physiological information collected in the field driving process. The main findings can be summarized as follows:

1. The proposed method can be used to estimate the DFD for road segments with rapid altitude change using the heart rate data collected from portable sensors.
2. The DFD increases the fastest at the road segment with constant and rapid altitude increases and vice versa. The DFD at the road segment with little altitude change also represents an S-shape curve.
3. The proposed method was only tested at the road segment with an altitude range from 3540 to 4767 m. More data are needed to verify the DFD estimation method at the road segment with another altitude level of the plateau.

## Figures and Tables

**Table 1:** Significance F and F values calculated by various time intervals.

Time Interval	30 s	1 min	2 min	3 min	4 min	5 min
Sig. F	0.009	0.011	0.012	0.022	0.103	0.142
F	112.6	82.2	54.7	45.3	38.9	21.8

**Table 2:** Results of F-test between two HRV-based algorithms and blinking frequency-based algorithm.

Statistical Indicators	Upslope Road Segment		Downslope Road Segment	
	Blinking-DFD	Blinking-rDFD	Blinking-DFD	Blinking-rDFD
Sum of Squares	10,447.116	4853.339	8418.968	4607.189
F	26.402	20.018	20.477	17.206
Sig. F	0.082	0.021	0.091	0.013
R^2^	0.199	0.965	0.296	0.938
